# Three Months of High-Fructose Feeding Fails to Induce Excessive Weight Gain or Leptin Resistance in Mice

**DOI:** 10.1371/journal.pone.0107206

**Published:** 2014-09-11

**Authors:** Erik J. Tillman, Donald A. Morgan, Kamal Rahmouni, Steven J. Swoap

**Affiliations:** 1 Department of Biology, Williams College, Williamstown, Massachusetts, United States of America; 2 Department of Pharmacology, University of Iowa Carver College of Medicine, Iowa City, Iowa, United States of America; Hosptial Infantil Universitario Niño Jesús, CIBEROBN, Spain

## Abstract

High-fructose diets have been implicated in obesity via impairment of leptin signaling in humans and rodents. We investigated whether fructose-induced leptin resistance in mice could be used to study the metabolic consequences of fructose consumption in humans, particularly in children and adolescents. Male C57Bl/6 mice were weaned to a randomly assigned diet: high fructose, high sucrose, high fat, or control (sugar-free, low-fat). Mice were maintained on their diets for at least 14 weeks. While fructose-fed mice regularly consumed more kcal and expended more energy, there was no difference in body weight compared to control by the end of the study. Additionally, after 14 weeks, both fructose-fed and control mice displayed similar leptin sensitivity. Fructose-feeding also did not change circulating glucose, triglycerides, or free fatty acids. Though fructose has been linked to obesity in several animal models, our data fail to support a role for fructose intake through food lasting 3 months in altering of body weight and leptin signaling in mice. The lack of impact of fructose in the food of growing mice on either body weight or leptin sensitivity over this time frame was surprising, and important information for researchers interested in fructose and body weight regulation.

## Introduction

Concurrent with the rise in obesity in the United States since 1980, there has been a rise in food manufacturers' use of high fructose corn syrup as a low-cost sweetening ingredient, as well as an increase in total national sugar consumption through both food additives and sweetened beverages [1]–[3]. This observation has led to recent research into the consequences of over-consuming sugars, with a particular focus on fructose [4], [5]. Consumption of calorically sweetened beverages is correlated with childhood and adolescent obesity, increased abdominal adiposity, dyslipidemia, and elevated blood pressure [6]–[8]. In addition, fructose in particular causes decreased insulin sensitivity with elevated insulin levels [9].

Fructose has been suggested to be an obesogen [5], [10]. The consumption of dietary fructose in humans leads to significantly lower circulating glucose levels and higher triglyceride levels postprandially than dietary glucose, and the activity of the anorexigenic hormone leptin depends on levels of circulating glucose [11]. Therefore, one mechanism by which fructose may cause obesity is by failing to elevate glucose levels in the blood to the same levels as other dietary sweeteners after a meal, thereby failing to elicit the same leptin-mediated anorexia. Supporting its putative role in regulation of caloric intake, it is thought that fructose does not cause the satiety that is observed with other carbohydrates [12], [13]. Such a hypothesis is supported by recent work showing that glucose, but not fructose, ingestion reduces activation of the appetite-sensing region of the brain in humans and induces a network response consistent with satiety [14].

In particular, fructose is known to alter leptin sensitivity in rats [15]. Leptin is a canonical regulator of energy balance through its signaling in the arcuate nucleus of the hypothalamus, as well as other structures throughout the brain [11], [16]–[18]. Leptin reflects the general adiposity of an individual as it is secreted primarily by adipose tissue [19]. Leptin is anorexigenic, and as such, is part of a negative feedback system that decreases appetite as fat stores grow [16], [20], [21]. Deregulation of this system leads to obesity through several known mechanisms [22]–[25].

Fructose feeding has been implicated in the development of leptin resistance and obesity in numerous studies using rodents [15], [26]. Consumption of high fructose corn syrup administered in drinking water leads to obesity marked by increased body weight, body fat, and circulating triglycerides in rats [10]. Also in rats, the administration of a diet high in fructose diminishes leptin sensitivity and makes the animals susceptible to obesity, confirmed through increased weight gain when transferred to a high fat diet [15]. Rats on a high-fat, high-fructose diet that are switched to a high-fat, fructose-free diet become resensitized to leptin, centrally and peripherally, indicating that any induced leptin sensitivity is reversible simply by dietary changes [27]. Additionally, a sustained fructose diet led to oxidative stress, glucose intolerance, and insulin resistance in rats [28]. Together, these studies support the deleterious effects of dietary fructose in rodents.

Because of the numerous genetically modified strains available, mice represent a good model for studying genetic and nutritional interactions in obesity. We hypothesized that fructose feeding in young mice weaned directly to a diet containing high fructose would recapitulate the studies previously described in rats [15], [26], [29], [30]. Surprisingly, however, we found that 14 weeks of high fructose feeding in young C57Bl/6 mice did not negatively affect either their final body weight or sensitivity to leptin.

## Materials and Methods

### Animals – experiment #1

Pregnant C57Bl/6H females were purchased from Harlan and housed at Williams College, Williamstown, MA. Upon delivery, litters were housed with mothers for 3 weeks. 38 males were weaned from their mothers, and moved to individual housing at 23°C on a 12∶12-hour light-dark cycle and placed on one of two randomly assigned experimental diets. Control mice (n = 19) were allowed free access to a fructose-free diet (Harlan Teklad, TD.05075). This diet consisted of 5.2% fat (by weight) obtained from lard, 60% carbohydrates from corn starch, and 18.3% protein (mostly casein), with a caloric content of 3.6 kcal/g. The experimental group (n = 19) was given free access to a 60% fructose diet (Harlan Teklad, TD.89247), which is identical to the fructose-free diet with the exception that fructose was the carbohydrate, and not corn starch. Food intake was measured five times weekly and body weight twice weekly for 14 weeks in the hour before the onset of the dark phase. All procedures and experimental protocols were approved by the Williams College Animal Care and Use Committee.

### Animals – experiment #2

Male C57Bl/6J mice were purchased from The Jackson Laboratory and housed at the University of Iowa College of Medicine, Iowa City, Iowa. Upon delivery, mice were group housed (3 or 4 per cage) at 22–24°C on a 12∶12-hour light-dark cycle, and placed onto one of four randomly assigned experimental diets purchased from Research Diets Inc.: control diet (Catalog #D12450K: 12% of calories from fat, 67% carbohydrates, 21% protein, 3.9 kcal/g: n = 48), high fat diet (Catalog #D12451: 45% of calories from fat, 35% carbohydrates, 20% protein, 4.7 kcal/g: n = 56), a high sucrose diet (Catalog #D02022703: 60% of calories from sucrose, 13% fat, 27% protein, 3.9 kcal/g: n = 82), and a high fructose diet (Catalog #D02022704: 60% of calories from fructose, 13% fat, 27% protein, 3.9 kcal/g: n = 64). Body weight was measured once weekly for 20 weeks. This experiment was approved by the University of Iowa Animal Research Committee.

### Metabolic Rate Measurements

In weeks 2, 9, and 14 of high-fructose or control feeding of experiment #1, seven animals from each feeding group were housed for indirect calorimetry. The same seven animals were used each time metabolic rate was measured. To measure metabolic rate, mice were housed in standard, clear, polycarbonate mouse cages (volume approximately 7L) fitted with an airtight seal on the top, and inflow and outflow ports for air exchange. Room air was dried with calcium sulfate and pumped into the cage at a regulated flow rate of 250–350 mL min^−1^. Outflow from the cage was pulled through an O_2_ analyzer (S-3A/II; AEI Technologies, Naperville, IL) and subsequently a CO_2_ analyzer (CD-3A; AEI Technologies) at a rate of 140 mL min^−1^. Up to 3 cages were serially sampled, with an empty cage serving as a reference. The outflow from each cage was sampled once every 8 minutes. Ambient pressure and temperature were concurrently measured electronically (APR-1 and C10T, Data Sciences International, St. Paul, MN). Data from gas sensors were digitized and recorded using LabVIEW 8 (National Instruments, Austin, TX), while ambient pressure and temperature data were recorded using Dataquest A.R.T. 3.01 acquisition and analysis software (Data Sciences International, St, Paul, MN).

### Leptin Responsiveness

Recombinant murine leptin (R & D Systems), reconstituted in 20 mM Tris-HCl, pH 8, was administered to randomly chosen mice from experiment #1 after 14 weeks of either high-fructose (n = 9) or control (n = 10) diet intraperitoneally (I.P.) at a dose of 5 mg/kg of body weight, in a total volume of 0.1–0.2 ml. As vehicle control, 20 mM Tris-HCl, pH 8, was injected I.P. in the remainder of the high-fructose-fed (n = 10) or control-fed (n = 9) mice. Injections took place in the hour before the start of the dark phase. Food intake was recorded 6, 24, and 48 hours after injections.

### Plasma Analysis

At least 7 days after mice were tested for leptin sensitivity, while still on their respective diets, and one hour before the start of the dark phase, mice were sacrificed under isofluorane anesthesia and blood was collected by cardiac puncture. Plasma was isolated by microcentrifugation and immediately frozen at −80°C. Plasma non-esterified fatty acids were measured using the NEFA HR(2) colorimetric assay (Wako) following the microtiter procedure on a 96 well plate. Plasma triglyceride and glucose levels were measured using the LiquiColor Triglyceride assay and the LiquiColor Glucose assay (Stanbio).

### Data Analysis

Data are reported as mean ± standard error of the mean. Body weight averages and daily food intake for mice across feeding groups in experiment #1 were compared using a 2 (Treatment) × 14 (Week) ANOVA followed by a Bonferroni post hoc analysis. Total food consumption was compared using a non-paired T-test. Metabolic rate was compared across feeding groups using a 2 (Treatment) × 3 (Week) ANOVA followed by a Bonferroni post hoc analysis. Average food intake following leptin or saline injection was compared using an ANOVA followed by a Bonferroni post hoc analysis. Body weights for experiment #2 were compared across feeding groups using a 4 (Treatment) × 14 (Week) ANOVA followed by a Bonferroni post hoc analysis. For all tests, the significance threshold was p<0.05.

## Results

### Fructose feeding increases food intake and metabolic rate without increasing body weight

**Figure 1:**
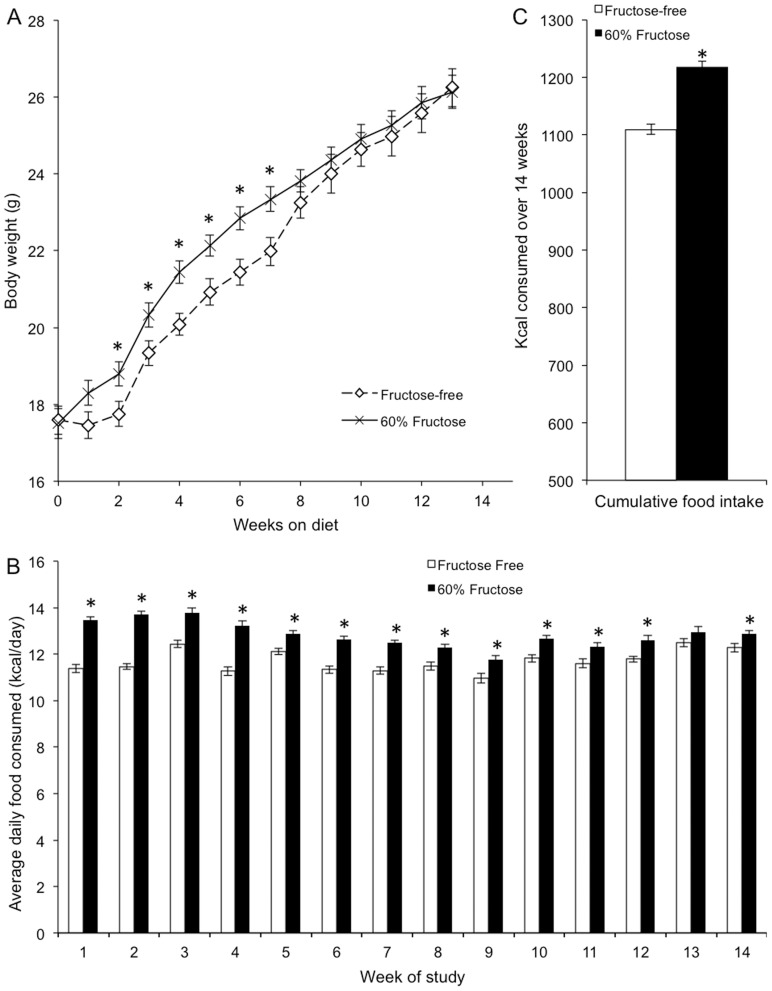
High-fructose-feeding does not increase body weight despite increased caloric intake. A) Transient changes in body weight in response to a high fructose diet. Fructose-fed mice were significantly heavier than control-fed mice from weeks 2–7 (*p<0.05). From 8 weeks to 14 weeks on their respective diets, there was no statistical difference in body weight between control-fed (n = 19) and fructose-fed (n = 19) mice. B) Fructose-fed mice consumed more food on average daily than control-fed mice for all weeks except week 13 (*p<0.05). C) Elevated cumulative caloric intake of fructose-fed mice. Over the course of the 14 weeks, fructose-fed mice consumed more total kcal than control-fed mice (*p<0.05).

At the beginning of experiment #1, the body weight of the male C57Bl/6H mice did not differ between the two groups (Control: 17.6±0.5 g, High fructose: 17.5±0.4 g). Statistical analysis revealed a significant main effect of time and diet, as well as a significant interaction. Analysis of the simple main effects indicated differences between high-fructose and control fed mice for weeks 2–8 (p<0.05; Figure 1A). After 14 weeks, mice on the high-fructose diet (26.1±0.4 g) did not have body weights that significantly differed from mice on the control diet (26.2±0.5 g) (Figure 1A).

The high-fructose fed mice consumed significantly more calories than control fed mice (Figure 1B). Statistical analysis revealed a significant main effect of time and diet, as well as a significant interaction. Analysis of the simple main effects indicated differences between high-fructose and control fed mice for all weeks except week 13 (p<0.05; Figure 1B). During week 2, for example, the control-fed mice consumed 80.3±1.0 kcal, significantly less (p<0.05) than 96.0±1.0 kcal consumed by the fructose-fed mice. By the end of the 14 weeks (Figure 1C), mice on the high-fructose diet had consumed 1218.9±9.3 kcal cumulatively, significantly more than mice on the control diet (1109.6±8.6 kcal).

**Figure 2:**
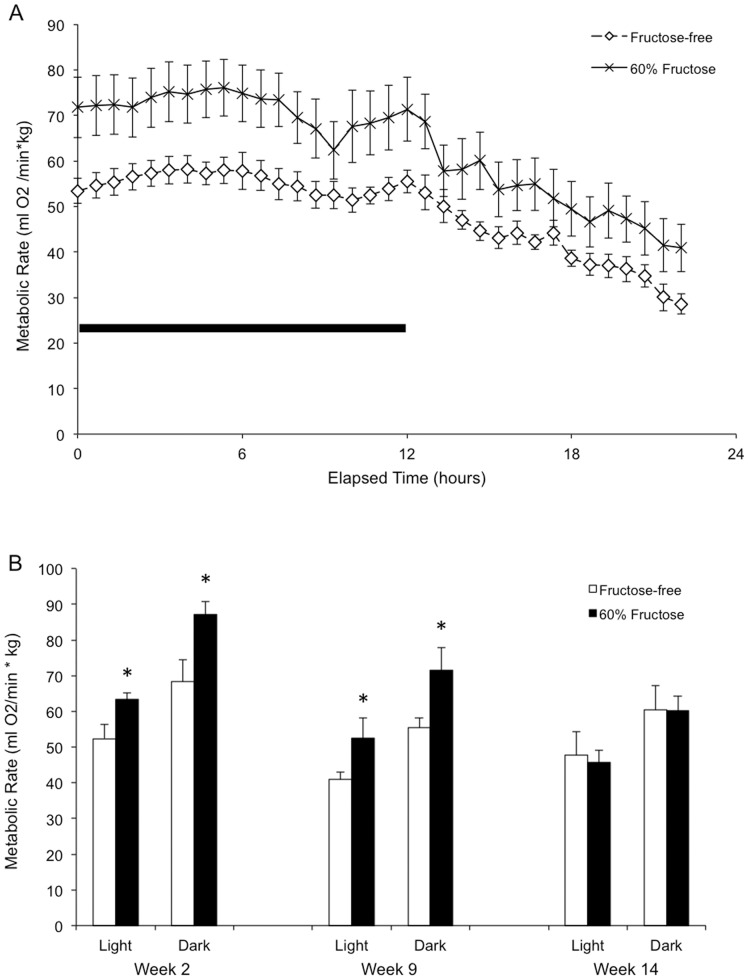
Differences in metabolic rate across feeding groups. A) Representative comparison of metabolic rates between high-fructose-fed (n = 7) and control-fed (n = 7) mice, week 9. Each data point represents a condensed 40-minute average for all the mice in that feeding group. The dark (active) phase—denoted by the black bar—spans hours 0–12, and the light (inactive) phase, hours 12–23. B) Average light- and dark-phase metabolic rates throughout the study. In weeks 2 and 9, fructose-fed mice (n = 7) had elevated metabolic rates compared to control-fed mice (n = 7). This difference was not present in week 14 (*- p<0.05 vs. control diet for indicated week).

Energy expenditure was estimated at three time points (week 2, week 9, and week 14) during the feeding experiment from 24-hour O_2_ consumption and CO_2_ production measurements. The respiratory exchange ratio was approximately 1.1–1.2 for both groups, and did not change with time (data not shown). Figure 2A shows metabolic differences between mice on the high-fructose diet and mice on the control diet during week 9. During both the dark phase and the light phase, fructose-fed mice consumed more O_2_ than their control-fed counterparts. This difference appeared greater in the dark phase (Figure 2B). Assuming the mice were oxidizing 100% carbohydrates, which is a reasonable assumption given the measured RER, the fructose-fed mice had a daily expenditure of 10.6±0.9 kcal/day during week 9, while the control-diet mice had a significantly lower expenditure at 8.4±0.4 kcal/day. It is notable that the metabolic rate of both groups of mice was lower than the caloric intake over the same time period (Figure 1B). When compared across weeks over the course of the study, we found this difference in caloric expenditure between the feeding groups was present in weeks 2, and 9, but not week 14 (Figure 2B).

### Fructose feeding does not change leptin sensitivity in mice

**Figure 3:**
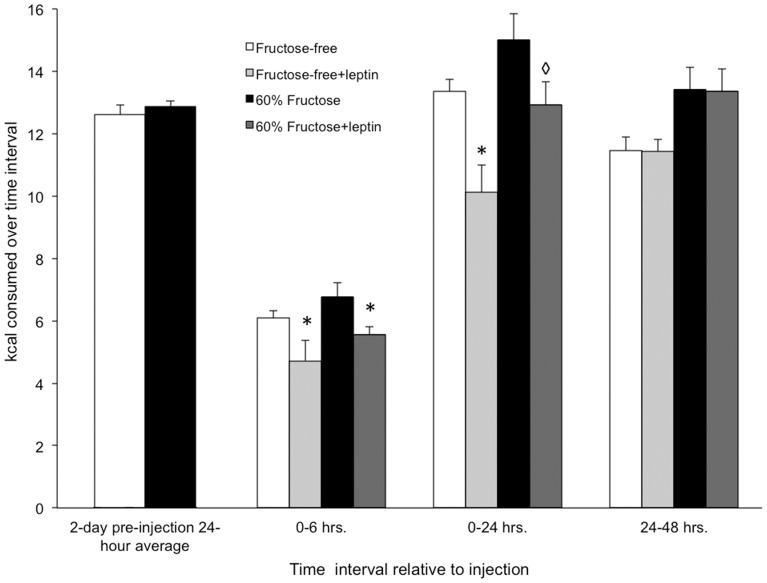
Persistence of leptin sensitivity in fructose-fed mice. Leptin injection leads to decreased food intake in both control-fed and fructose-fed mice compared to injection of vehicle over 6 and 24 hours, and returns to normal after 24 hours (*p<0.05 vs. the same diet and same time point with injection of vehicle; ◊p = 0.053 vs. 60% fructose). The first time point is a 24-hour average of the two days prior to the injection. Later time points are total caloric intake over indicated time interval after injection.

Despite the lack of difference in body weight between the two groups, we tested whether the fructose feeding could still induce changes in leptin sensitivity. Caloric intake (Figure 3) was measured for the two days prior to injection of either vehicle or leptin, and a 24-hour average caloric intake was calculated for mice in each feeding group. Control-fed mice consumed 12.6±0.3 kcal/day, and fructose-fed mice consumed 12.9±0.2 kcal/day during the two days prior to injection. Leptin-treated mice consumed significantly less food compared to vehicle injection after 6 hours for both control-fed (4.7±0.6 kcal vs. 6.1±0.2 kcal; p<0.05) and fructose-fed (5.5±0.3 kcal vs. 6.8±0.5 kcal; p = 0.050) mice. After 24 hours, mice consuming the control diet still had a significantly lower cumulative caloric intake in response to the leptin injection compared to vehicle injection (10.1±0.9 kcal vs. 13.4±0.4 kcal; p<0.05) but leptin administration to fructose-fed mice led only to a downward trend in caloric intake in the 24 hours post-injection compared to vehicle injection (12.9±0.8 kcal vs. 15.0±0.9 kcal; p = 0.053). Caloric intake in the second 24-hour interval after leptin injection was not different from vehicle injection for either control-fed (11.7±0.4 kcal vs. 11.9±0.3 kcal) or fructose-fed mice (13.1±0.5 kcal vs. 13.2±0.4 kcal).

### Fructose feeding does not alter circulating glucose, NEFAs, or triglycerides

Mice from both feeding groups were sacrificed 1 hour prior to the onset of the dark phase, and plasma was isolated. Plasma glucose, free fatty acid, and triglyceride levels were unchanged by the diet (Table 1).

**Table 1:** Fed plasma glucose, triglyceride, and free fatty acid concentrations.

	Control diet	High fructose diet
[Glucose] (mg/dL)	81.4±8.2	81.0±5.4
[Triglycerides] (mg/dL)	23.6±3.9	20.0±1.4
[NEFA] (mM)	0.42±0.05	0.36±0.04

### Absence of fructose-induced weight gain is not substrain specific

**Figure 4:**
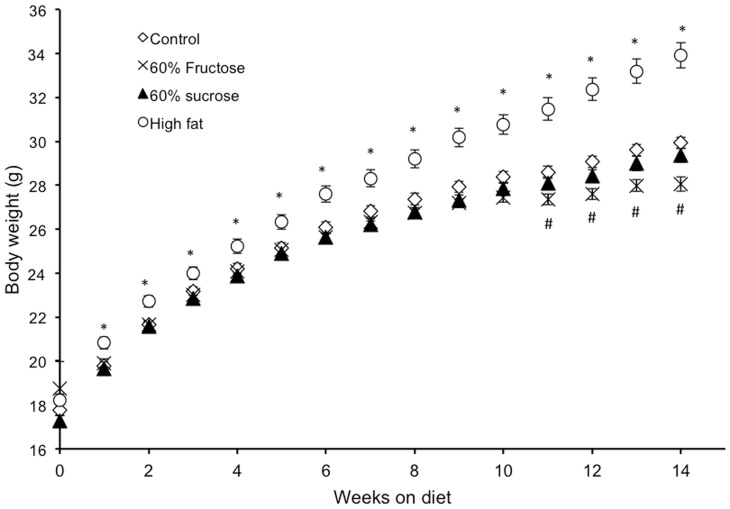
High-fat-feeding, but not high-fructose-feeding, increases body weight in C57Bl/6J mice. Male C57Bl/6J mice were fed a high fat (n = 56), high sucrose (n = 82), high fructose (n = 64), or control (n = 48) diet. The body weight of only the high-fat fed mice was significantly different from control-fed mice throughout the duration of the experiment (*p<0.05 vs. control diet and vs. high fructose diet and vs. high sucrose diet; #p<0.05 vs. control diet and vs. high sucrose diet).

It has become apparent that different strains of mice can respond differently to varying environmental conditions [31], [32]. Further, even the same strain purchased from different vendors (i.e. different substrains) can exhibit different phenotypes or drug sensitivities [33]–[35]. While C57BL/6 mice from Harlan were used for experiment #1 (C57Bl/6H), C57Bl/6 mice from Jackson labs was used in experiment #2 (C57Bl/6J). Here, mice that were the same age as in experiment #1 were fed one of four diets: 1) control, 2) high fat (45%), 3) 60% sucrose, which is a 30% fructose diet, and 4) 60% fructose. This substrain of mice (C57Bl/6J) gained significantly more weight than any of the other three groups when fed a high fat diet (Figure 4). The high fat diet had a greater caloric density at 4.7 kcal/gram of food than any of the other diets which each had a caloric density of 3.9 kcal/gram of food. Mice on either the 60% sucrose or 60% fructose did not exhibit any difference in body weight from control-fed mice until the 11^th^ week of treatment, when the body weights of the fructose group became significantly less than the 60% sucrose group or the normal chow (Figure 4). An additional 6 weeks, for a total of 20 weeks on these diets, produced no change in the relationship between body weights for any of the groups (high fat  = 37.2±0.7, 60% sucrose  = 30.8±0.4, 60% fructose  = 28.9±0.5, control  = 32.1±0.3 g). These data support the conclusion that in mice, fructose feeding does not induce excess weight gain over a span from 3–4.5 months, and prolonged exposure to a high-fructose diet can lead to a lower body weight.

## Discussion

After 14 weeks on a diet in which 60% of caloric intake through food was fructose, developing male mice failed to exhibit elevated body weight compared to control mice fed a diet lacking fructose (Figure 1 and 4). This was shown at two different institutions using C57Bl/6 mice from two different vendors (Harlan and Jackson Labs). In fact, the only long term weight gains seen in the C57Bl/6 mice were those on a high fat diet (Figure 4). Additionally, our results fail to support the hypothesis that fructose feeding for 3 months desensitizes mice to exogenous, systemically administered leptin. Indeed both control- and fructose-fed mice decreased caloric intake after leptin treatment, consistent with maintained leptin sensitivity despite a high-fructose diet. In response to fructose feeding for 3 months, we observed no changes in circulating non-esterified fatty acids, blood glucose, or blood triglycerides.

Based on previous studies in rodents, we had expected to see a drastic difference in body weight, leptin sensitivity, circulating triglycerides and circulating glucose between the feeding groups of mice [10], [13], [15], [26], [29], [36]–[39], [51]. There are at least three possibilities that could explain the lack of obesity in the mice on the high-fructose diet in the current study. First, many previous studies examining the effects of fructose consumption in rodents have utilized rats, which may differ in their metabolic response to fructose from the mice used in the current study. As mice have a much greater surface area to volume ratio than rats, the higher mass-specific metabolic rate of mice might allow for greater tolerance to fructose consumption as the fructose may be oxidized to CO_2_ and H_2_O to a greater extent in mice than rats, without the deleterious effects of fructose metabolites shuttled into VLDL (very low density lipoprotein) synthesis. However, it is important to note that wild-caught spiny mice actually lose weight on a 50% sucrose diet (25% of calories from fructose) that was associated with hepatomegaly, elevated VLDL, and β-cell hypertrophy [37]. Similarly, it was recently reported that mice on a high-fructose diet develop hepatic steatosis [40]. Among different strains of rats, there is variation in the phenotypic response to a high-fructose or sucrose diet. Some strains of rats gain weight on a high fructose diet [10], [15], [38], [39], some have no change in weight [26], [29], and one strain loses weight [39]. We found here that one substrain of mice (C57Bl/6H) on a high-fructose diet experienced a transient increase in body weight during development while a second substrain (C57Bl/6J) on a high-fructose diet failed to grow at the same rate as compared to siblings on a normal diet after 11 weeks of exposure.

A second reason for the lack of response to fructose may be age-dependent. In this experiment, we wished to test the effects of a high-fructose diet on the adolescent/young adult mouse, as fructose consumption appears to have an impact on adolescent humans [6], [41], [42]. The mice were weaned directly onto a high-fructose diet. In those studies that have utilized mice to examine the physiological changes induced by fructose consumption, they have done so using adult mice. It may be that the elevated mass-specific metabolic rate during maturation of the mouse in the current study was able to fully oxidize the fructose. However, multiple studies using fructose consumption in mice have observed increased adiposity or insulin resistance [43]–[45]. It is important to point out that those studies delivered fructose in liquid form as opposed to the current study, which delivered fructose in the food. While we did not measure adiposity or insulin sensitivity in the current study, we found that circulating fats were normal in fructose-fed mice. Given that fats in the blood are associated with insulin resistance, and fasting blood glucose was normal in these mice, it may be that fructose-fed mice in this study were not insulin resistant, in contrast to the above-mentioned studies [43]–[45]. This leads to the third possible explanation of the lack of observed effect– the different modes of fructose delivery. Whereas Bocarsly et al. [10] administered different sugars in aqueous solution as a supplement to standard chow, we opted to deliver fructose in food rather than in liquid to better control and monitor caloric intake. Their study found that high fructose corn syrup supplementation led to obesity in mice, characterized by increased body weight, elevated circulating triglycerides, and increased adiposity. We reasoned that fructose would lead to a positive energy balance independent of mode of delivery, though it remains possible that in mice, only aqueous fructose elicits an obesogenic response. This very different response in mice to either fed fructose (no long lasting effects of fructose consumption) or given fructose in liquid (significant long lasting effects of fructose consumption) is extremely important to highlight, and requires further detailed examination.

While we saw no overall difference in energy balance between the feeding groups at the study's conclusion, we observed that mice on the high fructose diet consumed more calories early in the study, accompanied by an elevated metabolic rate, and increased growth as mice compared to the control fed mice. Such an increase is supported by previous studies correlating fructose consumption with increased food intake, via the rapid early steps in fructose metabolism causing—through multiple biochemical steps—to lower malonyl-CoA in the hypothalamus [46]. Between weeks 2 and 7 in experiment #1, the fructose-fed mice gained weight faster than the control-fed mice, possibly due to faster acclimation to the sweeter food as compared to the unsweetened control food fed to the control mice. Because the difference in body weight disappears after 8 weeks through the end of the study, there must be either an equal shift towards negative energy balance in the fructose fed mice, or a corresponding positive shift in energy balance in the control fed mice. Because caloric intake in the control mice did not increase over the course of the study, we would predict energy expenditure in the fructose fed mice to increase. A metabolic increase has not been reported previously in response to fructose feeding. Indeed, it was recently reported that mice fed a high-fructose diet actually have a lower metabolic rate than mice fed a control diet [51]. However, our data suggest that such an increase in metabolic rate plays a role in the maintenance of energy balance between the groups: high fructose fed mice ate more and had a higher metabolic rate. Such a phenomenon may be behaviorally driven, in that the sweetness of the food increases consumption, which consequently increases digestion and measured metabolic rate [47], though it is also possible that fructose consumption increases metabolic rate independent of the elevated metabolic rate associated with increased food intake. In addition, many hormones regulate diet-induced thermogenesis as well as cold-induced thermogenesis in mice [48]. Further research is warranted to examine the circulating levels of these hormones throughout exposure of mice to a high fructose diet. It should be noted, however, that the faster weight gain in fructose-fed mice was not observed in experiment #2.

At the conclusion of experiment #1, we observed no change in leptin sensitivity resulting from fructose feeding, in contrast to previous studies conducted in rats [15]. Fructose-induced leptin resistance had been observed accompanying increased triglycerides, which Shapiro et al. posited decreased the permeability of the blood-brain barrier (BBB) to leptin [49]. However, recent evidence suggests that the ability of leptin to cross the blood brain barrier is not impaired in fructose-fed rats [30]. Three months of fructose feeding failed to induce leptin resistance, changes in circulating glucose, or changes in circulating triglycerides/free fatty acids. As stated above, two studies have found increased insulin resistance in high-fructose fed mice [40], [43], [45], and one has shown decreased glucose tolerance [51]. The fructose-fed mice in the current study were not diabetic (Table 1), exhibited a normal body weight, and had normal circulating fats in the blood after 14 weeks on the diet. Because obesity can deregulate leptin signaling, an attractive explanation for our results is that a high-fructose diet in mice fails to cause obesity, and therefore no leptin resistance would be expected. A high fructose diet can lead to an increase in visceral adiposity with no change in body weight that might influence leptin or insulin sensitivity [51]. However, leptin resistance and obesity do not always go hand-in-hand; leptin resistance can occur in the absence of obesity. Despite the lack of effect of dietary fructose on obesity in these mice, we tested leptin sensitivity in fructose-fed mice and found they displayed normal sensitivity to leptin. It is possible that 3 months is not long enough to evoke changes in leptin sensitivity. Given the lack of effect on body weight after 4.5 months on a high fructose diet, the duration of a high-fructose diet may need to be considerably longer to induce obesity or leptin resistance. Similarly, it may be that our study started too late for such diet-induced phenotypic variation to manifest in mice, and perhaps the mothers should have been fed either high-fructose or control food while nursing, or during the course of their sexual maturation, as parental nutrition is known to play a role in epigenetic patterning of offspring [50].
